# Observations on the Topography, Climate, and Prevalent Diseases of the Island of Jersey

**Published:** 1837-10-01

**Authors:** 


### Observations on the Topography, Climate, and Prevalent Diseases of the Island of Jersey. By *G. S. Hooper*, M.D.

This little volume, which appears to have been carefully compiled, contains much that is merely of local interest to the inhabitants themselves, such as suggestions respecting improvements, &c. &c. Still it exhibits many facts and points of information not uninteresting to the medical profession of Britain generally. We shall endeavour to cull out this species of material from the volume for the benefit of our readers.

Jersey is the largest and most southern of the "Channel Isles," and has a superficies of about 40 thousand acres, variegated by hills and vales, the ground generally sloping down, however, towards the South. The soil is a deep sandy loam, with a sub-soil of clay, based on granitic rocks. The island is very fertile. In the inland or elevated parts of the island the water is very pure, and soft—in the lower parts of the island, the water contains more saline ingredients, and is rather hard, as well as turbid after rains, having a vapid taste, and disagreeable odour. The population is about 40,000—industrious—but not very temperate. Wine and spirits are too cheap!

The climate, as might be expected from the situation and small extent of the island, is "remarkably mild." It is, however, habitually damp, independently of the nature of the surface, and the quantity of rain that falls.

The mean temperature of the island at St. Helier's is 53°—the entire range of the mercury (in five successive years), being 62°—the two extremes 88° and 26°. August is the hottest, and January the coldest month. The mean variation between successive months is 3° 67′. There are some sudden variations of temperature. The mean daily range at St. Helier's, during five years' observation, was 11° 70′.

### Diseases of the Island.

Intemperance has already been hinted at.

"A very fertile source of disease, in this otherwise happy spot, arises from an excessive use of ardent spirits, the mean price of which is attended by the double evil of increasing temptation, and facilitating indulgence. Among the labouring classes, especially, this disgraceful and ruinous habit produces incalculable mischief. But this remark does not, I am sorry to say, apply to that portion of our little community only; and it is a melancholy truth, that superior education,—which ought to open the mind to a due sense of decorum, consequences, and moral obligations,—does not always effectually guard it against this debasing propensity. On the contrary, instances are not wanting, here, of men, who after an honorable and active life, find in the comfort and retirement formerly desired, and to which this favoured spot is so well adapted, nothing but monotony, and insufferable dullness. For the want of intellectual resources, they seek, by the stimulus of liquor, to dissipate the *ennui* which oppresses their vacant minds. And, even, when this dangerous practice does not draw such persons into habitual drunkenness, it most commonly forms an error in diet, the effects of which are, sooner or later, displayed in a variety of consequent disorders." 142.

This picture, we fear, applies to a large class of half-pay officers from both services, who find, in Guernsey and Jersey cheap eating and drinking—their chief solace after a life of activity—and when all means of employing either mind or body are sought in vain !

This island is not distinguished by the prevalence of any particular disease. The most frequent are—

" 1.—In *infancy*, catarrhal affections; remittent fever, often complicated by acute hydrocephalus. 2.—In *childhood*, catarrhal affections: subacute inflammation of the bowels. 3.—In *adolescence*, continued fever; pleurisy. 4.—In the *adult age*, gastric affections; bronchitis; rheumatism; chronic bronchitis; chronic pleurisy, with effusion; dilatation of the right cavities of the heart, without hypertrophy; ascites." 144.

The island presents, of course, a fair proportion of most other maladies, in consequence of the great number of strangers that are constantly sojourning in that place. It is said that scrofulous affections are more numerous in Jersey than in other parts of similar latitude; but this is doubted by our author. He avers, however, that although many of the exciting causes of phthisis, as inflammatory affections of the air-passages, are plentiful in the island, yet consumption itself is more rare than in England. And here the author takes up the subject of remedial agency of the Jersey climate. He has observed that tubercular consumption proceeds very slowly, in many cases,—so much so that doubts are occasionally entertained whether or not the disease be actual phthisis. This is the case with chronic diseases generally in the climate in question. Arguing from these facts, and from an observation of thirteen years' experience, he thinks he may fairly represent Jersey " as a sort of initiatory climate, for individuals, who, from a protracted sojourn in tropical latitudes, have been rendered unable to resist the inclemencies of less favoured situations."

" During nearly three-fourths of the year, the soft and equable character of this climate, joined to a moderate temperature in the two extremes, will generally, prove beneficial in the incipient stage of pulmonary consumption, when the subjects of that most fatal of all human diseases, have, hitherto, lived in less temperate regions. This affection is, most commonly, ushered in by a marked period of irritation, requiring depletion, and sedative remedies; both of which means will take greater, and more durable effects under our sky, than in situations subject to more extensive variations, and ranges of atmospheric heat. It would be difficult to understand upon what just grounds this climate could have been supposed unfavorable to invalids liable to hæmoptysis, unless it was in the last stage of tuberculous consumption. For the truth is, hæmoptysis, whether as an idiopathic disease, or as a complication of phthisis, is, here, comparatively rare, local agencies tending much more to prevent, than to induce a plethoric state of the system. And, indeed, many instances have come under my observation, of hæmoptysis having been effectually checked by a residence of one or more winters in this island, when no organic disease of the lungs existed, and when the hemorrhage was simply the result of local congestion." 190.

We have thus picked out the chief points of interest to the general professional reader. There are many topics of a local, topographical, and domestic nature which will be read with interest by those who reside in Jersey. In respect to the attractions which its climate may possess for phthisical invalids, we confess that we do not hold them in very high estimation. The fact of catarrhal and inflammatory affections of the chest being common in the channel islands, ought to make us pause, notwithstanding the ingenious arguments of our author, before we recommend any one labouring under hæmoptysis or tubercular disease to locate himself in Jersey. This and its sister isles, however, will ever present most potent attractions for all those who love a glass of grog and a bottle of wine for one third the price of those articles in England. The cheapness of living, too, must induce many families labouring under the "res angusta domi," to remove to a climate which is mild, and to a land where the necessaries of life are cheap.

---

